# Supplementary material for: Heparin-based hydrogel scaffolding alters the transcriptomic profile and increases the chemoresistance of MDA-MB-231 triple-negative breast cancer cells
Source: Biomater Sci. 2020 Feb 13;8(10):2786–96. doi: 10.1039/c9bm01481k (PMC7497406; doi:10.1039/c9bm01481k)
Supplement: Supplementary file 2 [file BM-008-C9BM01481K-s002.zip › Supplementary File 4/HP-BhydrogelvControl/Pathways/my_analysis.Gsea.1545201079907/HALLMARK_PEROXISOME.html]

Details for gene set HALLMARK\_PEROXISOME[GSEA]

|  || Dataset | expr.class.cls#HYD\_versus\_CONTROL.class.cls#HYD\_versus\_CONTROL\_repos |
| Phenotype | class.cls#HYD\_versus\_CONTROL\_repos |
| Upregulated in class | CONTROL |
| GeneSet | HALLMARK\_PEROXISOME |
| Enrichment Score (ES) | -0.39836848 |
| Normalized Enrichment Score (NES) | -1.6532899 |
| Nominal p-value | 0.0 |
| FDR q-value | 0.0075272974 |
| FWER p-Value | 0.03 |
Table: GSEA Results Summary

  

Fig 1: Enrichment plot: HALLMARK\_PEROXISOME      
 Profile of the Running ES Score & Positions of GeneSet Members on the Rank Ordered List

  

| PROBE | DESCRIPTION (from dataset) | GENE SYMBOL | GENE\_TITLE | RANK IN GENE LIST | RANK METRIC SCORE | RUNNING ES | CORE ENRICHMENT || 1 | MLYCD | na |  |  | 231 | 2.846 | 0.0332 | No |
| 2 | PEX6 | na |  |  | 1559 | 1.547 | -0.0115 | No |
| 3 | CTBP1 | na |  |  | 1775 | 1.456 | 0.0004 | No |
| 4 | CLN8 | na |  |  | 1993 | 1.366 | 0.0108 | No |
| 5 | ACAA1 | na |  |  | 3257 | 0.975 | -0.0398 | No |
| 6 | ERCC3 | na |  |  | 3600 | 0.894 | -0.0434 | No |
| 7 | HSD11B2 | na |  |  | 3749 | 0.858 | -0.0375 | No |
| 8 | HRAS | na |  |  | 3805 | 0.850 | -0.0268 | No |
| 9 | NUDT19 | na |  |  | 3883 | 0.837 | -0.0175 | No |
| 10 | CEL | na |  |  | 4492 | 0.719 | -0.0379 | No |
| 11 | ABCC5 | na |  |  | 4570 | 0.697 | -0.0308 | No |
| 12 | SIAH1 | na |  |  | 4698 | 0.675 | -0.0267 | No |
| 13 | DLG4 | na |  |  | 4990 | 0.628 | -0.0319 | No |
| 14 | ABCD1 | na |  |  | 5174 | 0.604 | -0.0319 | No |
| 15 | GSTK1 | na |  |  | 5693 | 0.526 | -0.0506 | No |
| 16 | ESR2 | na |  |  | 5860 | 0.505 | -0.0513 | No |
| 17 | CLN6 | na |  |  | 6680 | 0.395 | -0.0878 | No |
| 18 | SLC25A19 | na |  |  | 7240 | 0.333 | -0.1118 | No |
| 19 | SLC23A2 | na |  |  | 7458 | 0.312 | -0.1181 | No |
| 20 | MVP | na |  |  | 7798 | 0.273 | -0.1315 | No |
| 21 | ATXN1 | na |  |  | 8586 | 0.195 | -0.1696 | No |
| 22 | PEX14 | na |  |  | 9195 | 0.126 | -0.1994 | No |
| 23 | ECH1 | na |  |  | 9762 | 0.069 | -0.2279 | No |
| 24 | DHRS3 | na |  |  | 9773 | 0.068 | -0.2273 | No |
| 25 | FDPS | na |  |  | 10354 | 0.012 | -0.2575 | No |
| 26 | RETSAT | na |  |  | 10420 | 0.005 | -0.2608 | No |
| 27 | ABCB9 | na |  |  | 10786 | -0.036 | -0.2793 | No |
| 28 | FADS1 | na |  |  | 11338 | -0.094 | -0.3067 | No |
| 29 | CRAT | na |  |  | 11449 | -0.107 | -0.3107 | No |
| 30 | HSD3B7 | na |  |  | 11566 | -0.122 | -0.3148 | No |
| 31 | DHCR24 | na |  |  | 11725 | -0.142 | -0.3209 | No |
| 32 | PEX5 | na |  |  | 11901 | -0.163 | -0.3274 | No |
| 33 | TSPO | na |  |  | 12292 | -0.212 | -0.3444 | No |
| 34 | ACOT8 | na |  |  | 12300 | -0.213 | -0.3414 | No |
| 35 | SMARCC1 | na |  |  | 12750 | -0.252 | -0.3609 | No |
| 36 | ITGB1BP1 | na |  |  | 13226 | -0.314 | -0.3807 | No |
| 37 | ACSL5 | na |  |  | 13564 | -0.364 | -0.3926 | Yes |
| 38 | SOD2 | na |  |  | 13651 | -0.378 | -0.3911 | Yes |
| 39 | MSH2 | na |  |  | 13742 | -0.394 | -0.3895 | Yes |
| 40 | GNPAT | na |  |  | 13747 | -0.395 | -0.3834 | Yes |
| 41 | ACSL4 | na |  |  | 13801 | -0.403 | -0.3798 | Yes |
| 42 | ABCB4 | na |  |  | 13965 | -0.415 | -0.3817 | Yes |
| 43 | NR1I2 | na |  |  | 13986 | -0.415 | -0.3761 | Yes |
| 44 | HMGCL | na |  |  | 14016 | -0.419 | -0.3710 | Yes |
| 45 | IDH2 | na |  |  | 14279 | -0.459 | -0.3774 | Yes |
| 46 | CRABP2 | na |  |  | 14283 | -0.460 | -0.3702 | Yes |
| 47 | PEX2 | na |  |  | 14321 | -0.468 | -0.3647 | Yes |
| 48 | STS | na |  |  | 14380 | -0.474 | -0.3602 | Yes |
| 49 | ECI2 | na |  |  | 14523 | -0.495 | -0.3597 | Yes |
| 50 | ACOX1 | na |  |  | 14591 | -0.504 | -0.3552 | Yes |
| 51 | ACSL1 | na |  |  | 14604 | -0.507 | -0.3477 | Yes |
| 52 | ABCD3 | na |  |  | 14612 | -0.508 | -0.3400 | Yes |
| 53 | FABP6 | na |  |  | 14803 | -0.538 | -0.3414 | Yes |
| 54 | CNBP | na |  |  | 14843 | -0.543 | -0.3348 | Yes |
| 55 | SLC35B2 | na |  |  | 15200 | -0.595 | -0.3439 | Yes |
| 56 | PEX11A | na |  |  | 15218 | -0.600 | -0.3353 | Yes |
| 57 | SEMA3C | na |  |  | 15278 | -0.613 | -0.3286 | Yes |
| 58 | PEX11B | na |  |  | 15553 | -0.661 | -0.3324 | Yes |
| 59 | ISOC1 | na |  |  | 15866 | -0.717 | -0.3373 | Yes |
| 60 | TOP2A | na |  |  | 15987 | -0.741 | -0.3318 | Yes |
| 61 | SLC25A17 | na |  |  | 16046 | -0.759 | -0.3227 | Yes |
| 62 | YWHAH | na |  |  | 16078 | -0.765 | -0.3122 | Yes |
| 63 | LONP2 | na |  |  | 16328 | -0.816 | -0.3122 | Yes |
| 64 | SLC25A4 | na |  |  | 16335 | -0.818 | -0.2995 | Yes |
| 65 | ERCC1 | na |  |  | 16348 | -0.820 | -0.2870 | Yes |
| 66 | RDH11 | na |  |  | 16414 | -0.835 | -0.2771 | Yes |
| 67 | IDI1 | na |  |  | 16426 | -0.838 | -0.2644 | Yes |
| 68 | CADM1 | na |  |  | 16551 | -0.866 | -0.2571 | Yes |
| 69 | BCL10 | na |  |  | 16727 | -0.907 | -0.2518 | Yes |
| 70 | PRDX1 | na |  |  | 16800 | -0.928 | -0.2408 | Yes |
| 71 | SCP2 | na |  |  | 16821 | -0.932 | -0.2270 | Yes |
| 72 | FIS1 | na |  |  | 17201 | -1.051 | -0.2301 | Yes |
| 73 | PRDX5 | na |  |  | 17276 | -1.076 | -0.2168 | Yes |
| 74 | PEX13 | na |  |  | 17316 | -1.089 | -0.2015 | Yes |
| 75 | VPS4B | na |  |  | 17352 | -1.102 | -0.1858 | Yes |
| 76 | HSD17B4 | na |  |  | 17589 | -1.191 | -0.1792 | Yes |
| 77 | EHHADH | na |  |  | 17881 | -1.324 | -0.1733 | Yes |
| 78 | IDE | na |  |  | 18010 | -1.389 | -0.1579 | Yes |
| 79 | ALDH9A1 | na |  |  | 18095 | -1.437 | -0.1394 | Yes |
| 80 | IDH1 | na |  |  | 18161 | -1.475 | -0.1193 | Yes |
| 81 | ELOVL5 | na |  |  | 18504 | -1.707 | -0.1100 | Yes |
| 82 | HSD17B11 | na |  |  | 18720 | -1.913 | -0.0908 | Yes |
| 83 | SOD1 | na |  |  | 18799 | -2.015 | -0.0628 | Yes |
| 84 | CAT | na |  |  | 19052 | -2.529 | -0.0357 | Yes |
| 85 | CDK7 | na |  |  | 19098 | -2.717 | 0.0052 | Yes |
Table: GSEA details [plain text format]

  

Fig 2: HALLMARK\_PEROXISOME      
 Blue-Pink O' Gram in the Space of the Analyzed GeneSet

  

Fig 3: HALLMARK\_PEROXISOME: Random ES distribution      
 Gene set null distribution of ES for **HALLMARK\_PEROXISOME**
